# Artificial intelligence-driven diagnosis of β-thalassemia minor & iron deficiency anemia using machine learning models

**DOI:** 10.5937/jomb0-38779

**Published:** 2024-01-25

**Authors:** Süheyl Uçucu, Fatih Azik

**Affiliations:** 1 Ministry of Public Health Care Laboratory, Department of Medical Biohemistry, Muğla, Turkey; 2 Muğla Sıtkı Koçman University, Faculty of Medicine, Department of Pediatric Hematology-Oncology, Muğla, Turkey

**Keywords:** beta-thalassemia minor, iron deficiency anemia, machine learning, artificial neural network, decision tree, beta-talasemija minor, anemija zbog nedostatka gvožđa, mašinsko učenje, veštačka neuronska mreža, način odlučivanja

## Abstract

**Background:**

Iron deficiency anemia (IDA) and b-thalassemia minor (BTM) are the two most common causes of microcytic anemia, and although these conditions do not share many symptoms, differential diagnosis by blood tests is a time-consuming and expensive process. CBC can be used to diagnose anemia, but without advanced techniques, it cannot differentiate between iron deficiency anemia and BTM. This makes the differential diagnosis of IDA and BTM costly, as it requires advanced techniques to differentiate between the two conditions. This study aims to develop a model to differentiate IDA from BTM using an automated machine-learning method using only CBC data.

**Methods:**

This retrospective study included 396 individuals, consisting of 216 IDAs and 180 BTMs. The work was divided into three parts. The first section focused on the individual effects of hematological parameters on the differentiation of IDA and BTM. The second part discusses traditional methods and discriminant indices used in diagnosis. In the third section, models developed using artificial neural networks (ANN) and decision trees are analysed and compared with the methods used in the first two sections.

## Abstract (sr)

**Uvod:**

Anemija zbog nedostatka gvožđa (IDA) i b-talasemija minor (BTM) su dva načešća uzroka mikrocitne anemije, i iako ova stanja nemaju mnogo simptoma, diferencijalna dijagnoza na osnovu testova iz krvi je dugotrajan i skup proces. CBC može da se koristi za dijagnozu anemije, ali bez naprednih tehnika ne može da razlikuje anemiju zbog nedostatka gvožđa i BTM. Ovo čini diferencijalnu dijagnozu IDA i BTM skupom, jer zahteva napredne tehnike za razlikovanje ova dva stanja. Ova studija ima za cilj da razvije model za razlikovanje IDA od BTM koristeći automatizovanu metodu mašinske metode koristeći samo CBC podatke.

**Metode:**

Ova retrospektivna studija je obuhvatila 396 osoba, od kojih su 216 imale IDA, a 180 BTM. Aktivnosti su bile podeljene u tri dela. Prvi deo se fokusirao na individualne efekte hematoloških parametara na diferencijaciju IDA i BTM. Drugi deo govori o tradicionalnim metodama i diskriminantnim indeksima koji se koriste u dijagnostici. U trećem delu se analiziraju modeli razvijeni korišćenjem veštačkih neuronskih mreža (ANN) i stabala odlučivanja i porede se sa metodama korišćenim u prva dva dela.

**Rezultati:**

Zaključci studije su predstavljeni u tri dela. Prvi deo rezultata sugeriše da su MCV i RBC najefikasniji prediktori diskriminacije između ova dva stanja. Drugi deo rezultata ukazuje da su efekti diskriminantnih indeksa na diferencijaciju BTM i IDA bili slični. Međutim, korišćenje G i K i RDWI umesto drugih diskriminantnih indeksa za BTM i IDA u velikoj meri povećava diferencijaciju. Treći deo rezultata otkriva da su modeli mašinske primene kao što je ANN bolji od tradicionalnih diskriminantnih indeksa.

**Zaključak:**

Ova studija preporučuje sistem zasnovan na veštačkoj neuronskoj mreži za razlikovanje ova dva stanja. U zaključku, naši rezultati pokazuju da ANN metoda funkcio niše bolje od postojećih metoda. Iako su drugi pristupi bili efikasni, veštačka inteligencija može bolje da predvidi prisustvo različitih varijanti hemoglobina od tradicionalnih statističkih pristupa. Ova diferencijacija je važna jer može da ima važne medicinske implikacije na negu pacijenata, planiranje porodice i genetsko savetovanje u vezi sa zdravljem. Model neuronske mreže takođe može da uštedi vreme, da košta manje i da lakše postavi dijagnozu.

## Introduction

According to the World Health Organization (WHO), iron deficiency anemia (IDA) is the world's most common type of anemia. A report by the WHO estimates that 33% of nonpregnant women, 40% of pregnant women, and 42% of children worldwide suffer from IDA [1] [2]. Although IDA generally presents with mild findings, it can cause complications such as impaired cognitive and motor functions, prenatal mortality, pregnancy-related maternal death, and cardiac failure, and may be associated with these diseases [1] [3]. Iron deficiency anemia (IDA) and b-thalassemia minor (BTM) are the two most common causes of hypochromic microcytic anemia, and although these conditions do not share many symptoms, the diagnosis by blood test is a time-consuming and expensive process [2]. CBC can be used to diagnose anemia, but without advanced techniques, it cannot differentiate between iron deficiency anemia and BTM [4]. This makes the differential diagnosis of IDA and BTM a costly procedure, as it requires advanced techniques to differentiate between the two conditions.

Beta thalassemia minor is one of the most common monogenetic diseases in the world, resulting from mutations in the beta-globin gene located on chromosome 11 [5] [6]. BTM is commonly asymptomatic. This condition, which is diagnosed after a complete blood count (CBC), HPLC, and hemoglobin electrophoresis analyses, may not be noticed until the mandatory premarital screening. However, HbA_2_ levels may appear lower, and BTM may be overlooked due to delta thalassemia, alpha thalassemia, preanalytic errors, and iron deficiency anemia [5] [7]. For this reason, misdiagnosis may cause unnecessary iron treatment and a loss of 2-3 months. In underdeveloped countries, this may even result in the birth of children with β-thalassemia major.

Considering the consequences of misdiagnosis of these two diseases, which are common worldwide, it is important to establish a new red blood cell indices-based screening model that is fast, inexpensive, highly accurate, and capable of automatic detection [2] [8]. This may prevent unnecessary treatment and the birth of homozygous individuals in the next generations. In addition, it saves time and medical costs.

The most common tests used to differentiate IDA and BTM are the hemoglobin (Hb), hematocrit (HCT), red blood cell (RBC), mean corpuscular volume (MCV), mean corpuscular hemoglobin (MCH), red cell distribution width (RDW), mean corpuscular hemoglobin concentration (MCHC), ferritin and hemoglobin A2 [1] [5]. These previous tests are used in various indices, such as the Mentzer index, Green & King index, England & Fraser index, red cell distribution width index, Ricerca index, Srivastava index, and Shine-Lal index, but these indices have relative successes (Table 1) [8] [9] [10] [11].

**Table 1:** Various indices used to differentiate IDA from BTM and their associated formulas [1]. MI, Mentzer Index; G & K, Green & King Index; E & F, England & Fraser Index; RDWI, Red cell distribution width index; RI, Ricerca Index; SI: Srivastava Index (SI); S & L, Shine & Lal Index.

Mentzer index (MI)	MCV/RBC
Green & King index (G & K)	MCV2 x RDW/100 x HGB
England & Fraser Index (E & F)	MCV- RBC-5Hb-3.4
RDW index (RDWI)	(MCVxRDW)/RBC
Ricerca index (RI)	RDW/RBC
Srivastava index (SI)	MCH/RBC
Shine & Lal index (S & L)	(MCV2 x MCH)/100

Machine Learning methods can produce fast and highly-accurate results with the ability to define problems, solve problems, and model nonlinear systems, which can process information in a complexity that the human mind cannot perceive. In recent years, machine learning (ML) methods, which is a type of artificial intelligence, have been used successfully in many fields around the world, from the classification of diseases to computational biology, from pharmacological research to bioengineering [12]. Artificial neural networks and deep learning algorithms, an ML method discovered inspired by the human brain's structure, can learn by itself, organise, link nonlinear relationships, and draw more specific results from the relationships between data [12] [13].

Artificial neural networks can provide us with more in-depth views of the differential diagnosis of IDA and BTM. In this study, we aimed to develop a fast and inexpensive automatic scanning model that can distinguish IDA and BTM from the relationships between hematological parameters using artificial neural networks.

## Materials and methods

Our study was carried out following the principles of the Declaration of Helsinki. This retrospective study included 205 (52%) female and 191 (48%) male patients aged between 18-65 years at Muğla Sıtkı Koçman University Training and Research Hospital between January 2015 and June 2021. The study was carried out in two groups. The first group consisted of 216 patients diagnosed with IDA between 01 January 2015 and 01 June 2021 at Muğla Sıtkı Koçman University Training and Research Hospital. The second group consisted of 180 patients diagnosed with BTM. The study protocol was approved by the local ethics committee of Muğla Sıtkı Koçman University with decision number 202/2021.

### Laboratory analysis process

The electrical impedance and optical scatter method determined red blood cell index parameters using Sysmex XN 1100 (Sysmex Diagnostic, Japan). Hemoglobin variant analysis was performed using the Primus Ultra II instrument (Trinity Biotech Diagnostic, Ireland) based on high-pressure liquid chromatography (HPLC). Serum iron and TIBC levels were measured by the photometric method on the Cobas 501 device (Roche Diagnostics, Germany). The ferritin level was analysed using the electrochemiluminescence method in the Cobas 601 device (Roche Diagnostics, Germany). Patients with the following conditions were excluded from the study: receiving iron treatment; having BTM; suffering from alpha-delta thalassemia; having thyroid disease; having high B12 or folate levels; having megaloblastic anemia; having systemic inflammation; and having gammo pathy.

### Model training and creation process

The Waikato Environment for Knowledge Analysis (WEKA, version 3.6.12, New Zeeland), an artificial intelligence program, was chosen for machine learning model training and data analysis. All rows having missing variables were removed from the dataset. Data were divided into training and validation sets, a standard practice in machine learning techniques. The selected training set is the data set used to train the model. Tenfold cross-validation (CV) was used to assess the robustness of the models. The models with the highest accuracy were selected. In artificial neural networks, we used the sigmoid activation function. On the other hand, for decision trees, the algorithm of choice was C4.5.

### Performance evaluation of the model validation process

The Waikato Environment for Knowledge Analysis (WEKA), an artificial intelligence program, was chosen for the training and data analysis of machine learning models. The performance of the model was evaluated according to; the sensitivity, specificity, negative predictive value (NPV), positive predictive value (PPV), false positive rate (FP), false negative rate (FN), true positive rate (TP), true negative rate (TN), per cent accuracy and F1 score.

### Statistical analysis

The statistical analyses were performed using the Jamovi (version 1.6.23 Jamovi Project, Sydney-Australia). Shapiro-Wilk test was used for normality testing. Differences between the groups are evaluated using Student's t-test or Mann-Whitney U test. ROC curve analysis was also performed to evaluate the performance of the discriminant indexes. An artificial neural network and decision tree models were made by using WEKA. For all statistical tests, P values 0.05 were considered significant.

## Results

Demographic data for BTM and IDA patients are presented in Table 2.

**Table 2:** Demographic data of BTM and IDA patients. Significant p < 0.05*

	BTM (n=180)	*IDA (n=216) *	*p*
	Mean ± SD	Mean ± SD	
Age (years)	34.4 ± 9.69	33.1 ± 9.73	0.140
Sex n (%)			
Female	86(48)	119(55)	< .001
Male	94(52)	97(45)	

The neural network architecture includes thirteen neurons in the input layer, two and three neurons in the hidden layers, and two neurons in the output layer. Thirteen inputs are fed into the network: Age; Sex; Hb, hemoglobin; RBC, red blood cell count; MCV, mean corpuscular volume; MCH, mean corpuscular hemoglobin; RDW, red blood cell distribution width; Iron; TIBC, total iron binding capacity; Ferritin; HbA2, hemoglobin A2; HbA0, hemoglobin A0.

**Figure 1:**
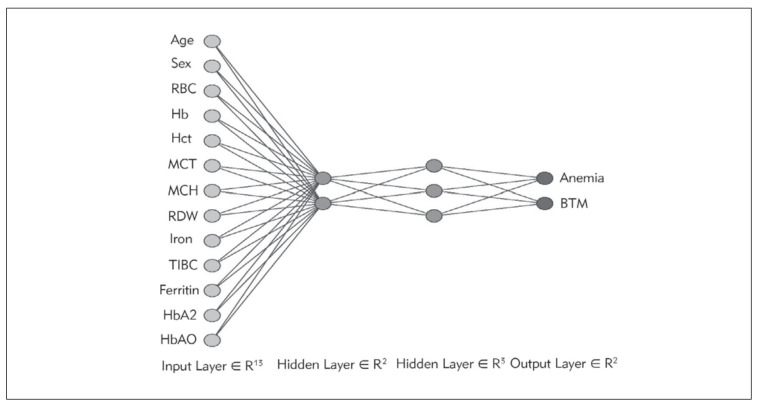
The architecture of the artificial neural network.

We trained an artificial neural network model with the training set, and it demonstrated high levels of sensitivity, specificity, accuracy, precision, and F1 score, all of which are critical indicators for distinguishing between IDA and BTM. Specifically, the model's sensitivity, specificity, accuracy, precision, and F1 score were 99.54%, 99.44%, 99.50%, 99.50%, and 99.50%, respectively (Figure 1, Table 3).

**Table 3:** Comparison of the diagnostic accuracy of discriminant indices, decision tree, and ANN models for discriminating IDA and BTM. NAUC, the area under the curve; PPV, positive predictive value; NPV, negative predictive value; FP, false positive; TP, true positive

Model	Accuracy-AUC	TP	FP	Sensitivity	Specificity	F1<br>score	PPV	NPV
Mentzer index (MI)	0.930	204	45	94.44	75	-	79.15	91.97
Green & King index (G & K)	0.974	211	17	97.69	90.56	-	92.54	97.02
England & Fraserindex (E & F)	0.970	209	31	96.76	82.78	-	87.08	95.51
RDW index (RDWI)	0.972	210	29	97.22	83.89	-	87.82	95.57
Ricerca index (RI)	0.957	203	31	93.98	82.78	-	86.75	91.98
Srivastava index (SI)	0.833	192	67	88.89	62.78	-	74.13	82.48
Shine & Lal index (S & L)	0.520	114	83	52.78	53.89	-	57.87	48.74
Decision tree	0.992	0.992	0.008	99.07	99.44	0.992	99.53	98.90
Artificial Neural Network (ANN)<br>with all parameters	0.995	0.995	0.005	99.54	99.44	0.995	99.54	99.44
Artificial Neural Network (ANN)<br>just CBC parameters	0.987	0.983	0.004	98.15	99.44	0.960	99.53	97.81
Artificial Neural Network (ANN)<br>just CBC without RBC	0.932	0.911	0.076	92.59	93.89	0.911	94.79	91.35
Artificial Neural Network<br>(ANN)just CBC without MCV	0.957	0.927	0.006	92.59	92.59	0.927	99.50	91.79

**Figure 2:**
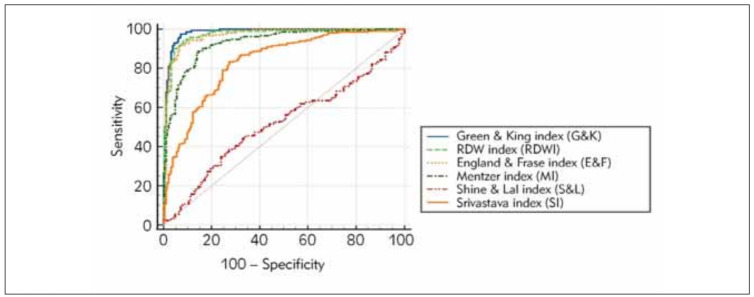
Comparison of diagnostic performance of discriminant indices with the ROC curve.

The findings from Figure 2 show that the Green & King index (G & K), England & Fraser index (E & F), and RDW index (RDWI) are all more effective predictors than other indices. These three indices had higher specificity, sensitivity, and area under the curve (AUC). Higher specificity, sensitivity and AUC levels indicate their ability to determine the desired outcome accurately.

According to Table 3, the most crucial difference in the differential diagnosis of IDA and BTM was the effect of RBC, which had a greater impact on the sensitivity, specificity, and F1 score of the diagnosis than MCV. This suggests that RBC is a more effective predictor than MCV in distinguishing between IDA and BTM.

Except for the Shine & Lal index and the Srivastava index, the effects of other discriminant indices in distinguishing between BTM and IDA (specificity, sensitivity, and AUC) were similar (Figure 2, Table 3-Table 4). However, using G & K and RDWI instead of other discriminant indices for BTM and IDA greatly increases differentiation. This shows that it is a more effective predictor than other discriminative indices (Figure 2, Table 3).

**Table 4:** Hematological variables and blood indices of the BTM and IDA groups. Ref: reference range; Hb: hemoglobin; RBC: red blood cell count; MCV: mean corpuscular volume; MCH: mean corpuscular hemoglobin; RDW: Red blood cell distribution width; Iron; TIBC: Total iron binding capacity; Ferritin; HbA_2_: Hemoglobin A2; HbA0: Hemoglobin A0; HCT: hemotocrit; Significant p < 0.05*

Parameters	BTM (n=180)		IDA (n=216)		p
	Median	IQR	Median	IQR	
RBC (10^12^/L)	6.13	5.58–6.59	4.84	4.52–5.15	< .001*
Hb (g/L)	1.22	1.13–1.35	0.93	0.84–0.10	< .001*
HCT (%)	39.1	36–42.7	31.7	29.5–34.0	< .001*
MCV (fL)	64.3	61.9–66.3	66.6	63.6–68.7	< .001*
MCH (pg)	20.1	19.3–21.0	19.1	18.0–20.4	< .001*
RDW (%)	17.3	16–18.3	19.0	17.8–20.3	< .001*
Serum Fe (μg/dL)	87.0	63–114	15	11.0–21.0	< .001*
TIBC (μg/dL)	323	299-357	429	400-455	< .001*
Ferritin (ng/L)	52.8	22.4–94.6	3.4	2.3–4.7	< .001*
Hb A2 (%)	4.3	4.07–4.6	1.6	1.50–1.70	< .001*
Hb A (%)	95.4	94.8–95.9	98.4	98.2–98.5	< .001*
Mentzer index (MI)	10.5	9.55–11.5	13.6	12.6–14.8	< .001*
Green & King index (G & K)	56	53.0–61.0	90	81.0–102	< .001*
England & Fraser Index (E & F)	-6.69	-13.0– -0.96	11.5	7.51–16.2	< .001*
RDW index (RDWI)	1.78	167-193	260	238-289	< .001*
Ricerca index (RI)	2.75	2.60–2.99	3.99	3.58–4.41	< .001*
Srivastava index (SI)	3.29	3.60–4.35	3.95	3.60–4.35	< .001*
Shine & Lal index (S & L)	838	749-920	854	722-958	0.516

## Discussion

This study showed that IDA and BTM could be separated with high accuracy using an automated machine learning method using only CBC data, and an artificial neural network-based system has higher diagnostic accuracy than discriminant indexes. In addition, the diagnostic power of the index parameters was compared and the individual effect of the hemogram parameters on the diagnosis was also examined.

This study evaluated the differential diagnosis of IDA and BTM in three parts. In the first section, the individual effects of hematological parameters were determined, how much they played in the differentiation of IDA and BTM, and which parameter was the most important was examined. The second part examines discriminant indices, which are the traditional diagnostic method. In the third part, ML models that make mathematical models from the relationships between the data, can learn three-dimensionally by themselves, create dynamics, and have the ability to draw more specific conclusions, and performance criteria are evaluated and compared with the methods in the first two sections [13].

According to the study results, among the hematological parameters, MCV and RBC are the most effective predictors in discriminating between IDA and BTM. MCV and RBC were found to have similar individual effects. It reduced the sensitivity and specificity in both parameters at a similar rate. Despite this similarity, the most important difference in the differential diagnosis of IDA and BTM was the effect of RBC, which had a greater impact on the sensitivity, specificity and F1 score of the diagnosis than MCV. This suggests that RBC is a more effective predictor than MCV in distinguishing between IDA and BTM (Table 3). Despite excluding these features from the dataset, ANN could distinguish both cases with high accuracy. In this way, it has been shown that ANN can only learn from the relationships between hematologic parameters in CBC and can distinguish both diseases from these relationships [4]. Many artificial intelligence studies have been conducted using various models and techniques for the differential diagnosis of BTM and IDA. In the study of Ayyıldız et al. [4], individual sensitivity of features, methodological performance, and gender effects were investigated. Following our study, they reported that the two most important tests in the diagnosis of BTM and IDA are MCV and RBC.

The most frequently used tests in the differential diagnosis of BTM and IDA in studies are RBC, MCV, MCH, RDW, HbA_2_, ferritin, and discriminant indexes [4]. These are indexes such as Mentzer index, Green & King index, England & Fraser index, RDWI. These indexes show that the most commonly used parameters are RBC and MCV [4]. To analyse the effects of these commonly used discriminant indices on the differentiation of BTM and IDA, we examined their diagnostic power and implications. The results of the study revealed that the Green & King index and the RDW index were more effective than other indexes in creating an accurate algorithm (Table 3) (AUC: 0.974, Sens: 97.69%, Spec: 90.56%). Hoffmann et al. [14] in a meta-analysis study in which they evaluated discriminant analyses in their study, reported the index with the highest discriminatory power as the M/H index. Urrechaga et al. [15] reported the best three indexes as the Green & King index, the RDWI index, and the Janel 11T score, which is consistent with our study [16]. However, they stated that many discriminant indexes are inaccurate or incomplete and even the index with the best performance is not good enough [9] [10] [16] [17]. In general, genetic and geographical differences may be a possible reason for the difference between studies and the indices not being sufficiently successful [9] [18]. Laengsri et al. [18] reported that ML techniques were able to distinguish with high success in their study consisting of 186 patients using ML techniques such as KNN, SVM, and ANN. Creating modern machine learning models naturally requires a high amount of data to achieve better results and training [18] [19]. Despite this, Laengsri et al. [18] have succeeded in the diagnostic performance of ML models.

According to the study results, the model with the highest performance among the ML models we trained was determined as ANN. ANN was able to distinguish 99.5% accuracy between the two conditions when compared to traditional methods. In the artificial neural network (ANN) model that we trained using only hemogram parameters, we achieved an area under the curve (AUC) of 0.987, and the F1 score was determined to be 0.96%. Our results show that our proposed method performs better than existing methods. Because our approach uses a neural network to predict hemoglobin variants more accurately, it can self-learn, associate nonlinear relationships, and draw more specific conclusions from relationships. AIl can predict the presence of various hemoglobin variants better than traditional statistical approaches, although other approaches are effective. It can be used to predict the presence of hemoglobin variants with greater success as the amount of data increases [13] [19]. Our approach not only outperforms discriminant indices, it can also provide shorter time, less cost, and easier diagnosis (Table 3, Figure 2).

## Conclusion

In conclusion, our results show that the ANN method performs better than the existing methods. Our approach is superior to existing methods because it uses a neural network to discriminate between the two conditions accurately. Although other approaches have been effective, artificial intelligence can better predict the presence of various hemoglobin variants than traditional statistical approaches. This differentiation is important because it can have important medical implications on patient care, family planning, and genetic counselling related to health. It can also save time, cost less, and make a more straightforward diagnosis.

## Dodatak

### Study limitations

We could not obtain reports on the genotypes of the patients in our study. We believe it would be better to use automatic programs that measure individual sensitivity since the high number of features can make training and optimisation difficult while creating ML models.

### Conflict of interest statement

All the authors declare that they have no conflict of interest in this work.
